# Feifukang ameliorates pulmonary fibrosis by inhibiting JAK-STAT signaling pathway

**DOI:** 10.1186/s12906-018-2297-3

**Published:** 2018-08-09

**Authors:** Hongbo Li, Zhenkai Wang, Jie Zhang, Youlei Wang, Chen Yu, Jinjin Zhang, Xiaodong Song, Changjun Lv

**Affiliations:** 1 Department of pulmonary medicine, School of Medicine, Shandong University, Jinan, 250100 Shandong China; 2 Department of pulmonary medicine, Binzhou Medical University Hospital, Binzhou, 256602 Shandong China; 3 Department of Cellular and Genetic Medicine, School of Pharmaceutical Sciences, Binzhou Medical University, No. 346, Guanhai Road, Laishan District, Yantai City, 264003 China

**Keywords:** Pulmonary fibrosis, Feifukang, Traditional Chinese medicine

## Abstract

**Background:**

Feifukang (FFK) is a traditional Chinese medicine composed of herbs that protect lung function. However, difficulty arises regarding the clinical application of FFK due to the complex mechanism of Chinese medicines. This study aimed to investigate the efficacy of FFK and explore its targeted genes and pathways.

**Methods:**

Histopathological changes and collagen deposition were measured to evaluate the effect of FFK on bleomycin-induced pulmonary fibrosis in mice. The differentially expressed targeted genes and pathways were first screened using RNA sequencing. Then network pharmacology and other experiments were conducted to confirm RNA sequencing data.

**Results:**

FFK treatment reduced the pathological score and collagen deposition, with a decrease in α-SMA and collagen. RNA sequencing and network pharmacology results all showed that FFK can ameliorate pulmonary fibrosis through multi-genes and multi-pathways. The targeted genes in JAK-STAT signaling pathway are some of the most notable components of these multi-genes and multi-pathways. Further experiments illustrated that FFK regulated phosphorylation of SMAD3, STAT3 and JAK1, and their co-expressed lncRNAs, which all are the important genes in JAK-STAT signaling pathway.

**Conclusion:**

FFK can ameliorate pulmonary fibrosis by inhibiting JAK-STAT signaling pathway and has potential therapeutic value for lung fibrosis treatment. Our study provides a new idea for the study of traditional Chinese medicine.

## Background

Pulmonary fibrosis occurs in various clinical settings and can be life threatening [1]; this disease is characterized by altered cellular composition and homoeostasis in peripheral lungs, thereby leading to excessive accumulation of extracellular matrix and loss of lung function [2]. In the past decade, researchers described several cellular and molecular signaling pathways implicated in the pathogenesis of pulmonary fibrosis; these results led to identification of new therapeutic targets. Many therapies are used in pulmonary fibrosis treatment; however, only a few can increase the survival rates and improve the quality of life of patients. Pirfenidone and nintedanib were approved for the treatment of pulmonary fibrosis [3, 4], but their side-effects, such as photosensitivity, gastrointestinal symptoms, and liver function test abnormalities, were often observed [5, 6]. This situation occurs because pulmonary fibrosis has complex regulatory networks that repress or induce the expression of a set of related target genes and pathways. Emerging evidence showed that genetic, epigenetic, and proteomic factors are involved in regulatory networks during development of pulmonary fibrosis. Instead of working through one pathway, these networks regulate the expression of entire sets of fibrosis-relevant genes by turning the pathways on or off [7, 8].

Chinese herbs exhibit low toxicity and no side effects for disease treatment. Therefore, traditional Chinese medicine (TCM) features the long history of disease treatment [9, 10]. However, the unclear mechanism of TCM has sparked criticism when this medicine has become more popular today [11, 12]. Thus, investigating the targeted genes and pathways is important to the modernization of TCM. Multiple approaches, including network pharmacology and pharmaco-genomics, have been utilized to investigate the mechanism of TCM. Qing-Luo-Yin and other TCMs have been studied by using these approaches [13–15]. As another novel high-throughput technique, RNA sequencing has become a potential research approach in disease treatment. However, this technology has not been popularized in TCM research.

Feifukang, also known as pulmonary rehabilitation mixture, comprises eight herbs including *Astragalus membranaceus* (Fisch) Bge., *Codonopsis pilosula* (Franch.) Nannf., *Ophiopogon japonicus*, *Schisandra chinensis*, *Panax notoginseng* (Burk.) F. H. Chen., *Bulbus fritillariae thunbergii*, *Rhizoma anemarrhenae*, and *Glycyrrhiza uralensis*, which is designed by our group based on clinical practice and drug screening for several decades. Through the experiment and the clinical test, FFK has been proven to have good curative effect for patients with pulmonary fibrosis. Our previous study demonstrated that FFK can prevent experimental pulmonary fibrosis in vitro and in vivo [16]. However, a critical issue must be addressed, namely, its mechanism of multi-genes and multi-pathways in treating pulmonary fibrosis. In the present study, we first combined RNA sequencing and network pharmacology to analyze the targeted multi-genes and multi-pathways of FFK in pulmonary fibrosis treatment. We hope provide the theoretical and experimental basis for the clinical application of FFK for lung fibrosis treatment. Meanwhile, we also hope to provide a new idea for the study of TCM.

## Methods

### Animal model and ethics statement

Eight-week-old C57BL/6 mice were obtained from the Model Animal Research Center of Nanjing University (Nanjing, China). All animal experiments were performed according to regulations established by the Ethics Committee on Animal Experiments of Binzhou Medical University (Approval number: No. 201704001). Mice were housed under a 12 h light/dark cycle and provided free access to food and water. Pulmonary fibrosis model was established as previously described [17]. Briefly, mice were administered with 5 mg/kg saline-dissolved bleomycin (BLM) via single intratracheal instillation under anesthesia. Sham control mice received an equal volume of saline only. On day 2, mice were randomly divided into the following groups (10 mice each): sham, BLM, and BLM + FFK-treated groups. FFK (3.0 g/kg) was administered orally once daily. Lungs of all mice were removed on day 28 for further analysis.

According to the 2013 AVMA Guidelines for the Euthanasia of Animals, intraperitoneal injection of ethanol is acceptable with conditions for use in animals [18]. Moreover, according to the method described by Allen-Worthington et al. [19], 70% (*v*/v) ethanol in 0.9% sterile saline was applied in the ventral chest region for getting deep anesthesia.

### Hematoxylin and eosin (H&E) and Masson’s trichrome staining

Histopathological changes and collagen deposition were assessed by the H&E and Masson staining, respectively. Lung tissues were fixed with 4% formalin overnight, dehydrated in 70% ethanol and cleared in xylene. Transverse sections of 4 um thickness were stained with H&E or Masson’s trichrome staining as previously described [17].

### Hydroxyproline content

Lung specimens were washed with saline and hydrolyzed with 0.6% hydrochloric acid at 100 °C for 5 h. Hydrolysates were neutralized with sodium hydroxide and diluted with distilled water. Hydroxyproline level in hydrolysates was colorimetrically determined by absorbance at 560 nm with p-dimethylaminobenzaldehyde and expressed as μg/mg wet tissue.

### Western blot

Twenty mircograms of protein sample was subjected to 10% sodium dodecyl sulfate polyacrylamide gel electrophoresis, transferred onto polyvinylidene difluoride membranes, and blocked with 7% non-fat milk in Tris-buffered saline and Tween-20 (TBST; 50 mM Tris-HCl [pH 7.6], 150 mM NaCl, 0.1% Tween-20). Membranes were washed thrice with TBST buffer and incubated at 4 °C overnight with specific antibodies. After washing with TBST, membranes were incubated with horseradish peroxidase-labeled IgG for 1.5 h. Membranes were then washed with TBST, incubated with ECL reagent, and exposed. Then, membranes were subsequently stripped and re-probed with glyceraldehyde 3-phosphate dehydrogenase antibody, which served as loading control.

### RNA-sequencing

A total amount of 2 μg RNA per sample was used as input material for the RNA sample preparations. Sequencing libraries were generated using NEBNext® Ultra™ RNA Library Prep Kit for Illumina® (#E7530L, NEB, USA) following the manufacturer’s recommendations and index codes were added to attribute sequences to each sample. Briefly, mRNA was purified from total RNA using poly-T oligo-attached magnetic beads. Fragmentation was carried out using divalent cations under elevated temperature in NEBNext First Strand Synthesis Reaction Buffer (5X). First strand cDNA was synthesized using random hexamer primer and RNase H. Second strand cDNA synthesis was subsequently performed using buffer, dNTPs, DNA polymerase I and RNase H. The library fragments were purified with QiaQuick PCR kits and elution with EB buffer, then terminal repair、A-tailing and adapter added were implemented. The aimed products were retrieved by agarose gel electrophoresis and PCR was performed, then the library was completed. RNA concentration of library was measured using Qubit® RNA Assay Kit in Qubit® 3.0 to preliminary quantify and then dilute to 1 ng/μL. Insert size was assessed using the Agilent Bioanalyzer 2100 system (Agilent Technologies, CA, USA), and qualified insert size was accurately quantified using StepOnePlus™ Real-Time PCR System (Library valid concentration>10 nM). The clustering of the index-coded samples was performed on a cBot cluster generation system using HiSeq PE Cluster Kit v4-cBot-HS (Illumina) according to the manufacturer’s instructions. After cluster generation, the libraries were sequenced on an Illumina Hiseq 4000 platform and 150 bp paired-end reads were generated.

### Quantitative real-time PCR (qRT-PCR)

Total RNA was isolated using TRIzol reagent (Invitrogen, Carlsbad, CA, USA). RNA quantity and quality were measured using the NanoDrop 2000 spectrophotometer (Thermo scientific, Waltham, USA) and RNA integrity was assessed by standard denaturing agarose gel electrophoresis. Complementary DNA synthesis was performed using the M-MLV reverse transcriptase kit (Invitrogen Carlsbad, CA, USA) following the manufacturer’s instructions. qRT-PCR was performed using a SYBR green-based PCR master mix kit (Takara, Shiga, Japan) on a Rotor Gene 3000 real-time PCR system from Corbett Research (Sydney, Australia).

### Analysis of network pharmacology

FFK comprises *Astragalus membranaceus* (Fisch) Bge., *Codonopsis pilosula* (Franch.) Nannf., *Ophiopogon japonicus*, *Schisandra chinensis*, *Panax notoginseng* (Burk.) F. H. Chen., *Bulbus fritillariae thunbergii*, *Rhizoma anemarrhenae*, and *Glycyrrhiza uralensis*. Active chemical components of these plants were collected and extracted from Chinese Pharmacopoeia 2015 edition, Web of Science, http://www.wanfangdata.com.cn/, http://www.cnki.net/, and www.ncbi.nlm.nih.gov/pubmed/. After reviewing databases, we extracted contents of various medicinal ingredients, and relevant activity reports were considered. Collected compounds included the main components of FFK. We encoded chemical compounds in the Traditional Chinese Medicine Systems Pharmacology database (http://sm.nwsuaf.edu.cn/lsp/index.php) to screen primary bioactive components using the absorption distribution metabolism excretion (ADME) system. Oral bioavailability (OB) totaled >10%, and drug likeness reached >0.04; these variables were used as thresholds for further extraction and optimization of medicinal ingredients. Molecules satisfying the criteria were used as bioactive compounds for further analysis.

Cytoscape is one of the most comprehensive tools for thorough analysis of biological networks. Various plugins extend functionality of Cytoscape by providing visualization and analysis of protein–protein interaction, gene regulation, gene co-expression, metabolism, signaling, and ecological networks as previously described [20, 21]. We extracted compounds covering primary drug-likeness component of FFK and encoded them into Cytoscape 3.2.1 software to construct a compound-medicine network. Subsequently, target bank, drug bank, binding DB, and *potential drug target database* were used to validate associative targets. Potential effective chemical compounds were inputted into the software to establish a compound-target network. The software was also used to construct a target-pathway network and explain target participation in pathways. Multiple targets indicated integral roles of FFK in IPF by sharing synergy targets of different compounds.

### Statistical analysis

Data were expressed as mean ± standard deviation (SD) of the indicated number of independent experiments. Statistical analyses was performed with SPSS 16.0 software using one-way analysis of variance and Student’s t-test. Statistically significant difference was considered at *p* < 0.05.

## Results

### Amelioration of FFK on BLM-induced pulmonary fibrosis in mice

**Fig. 1:**
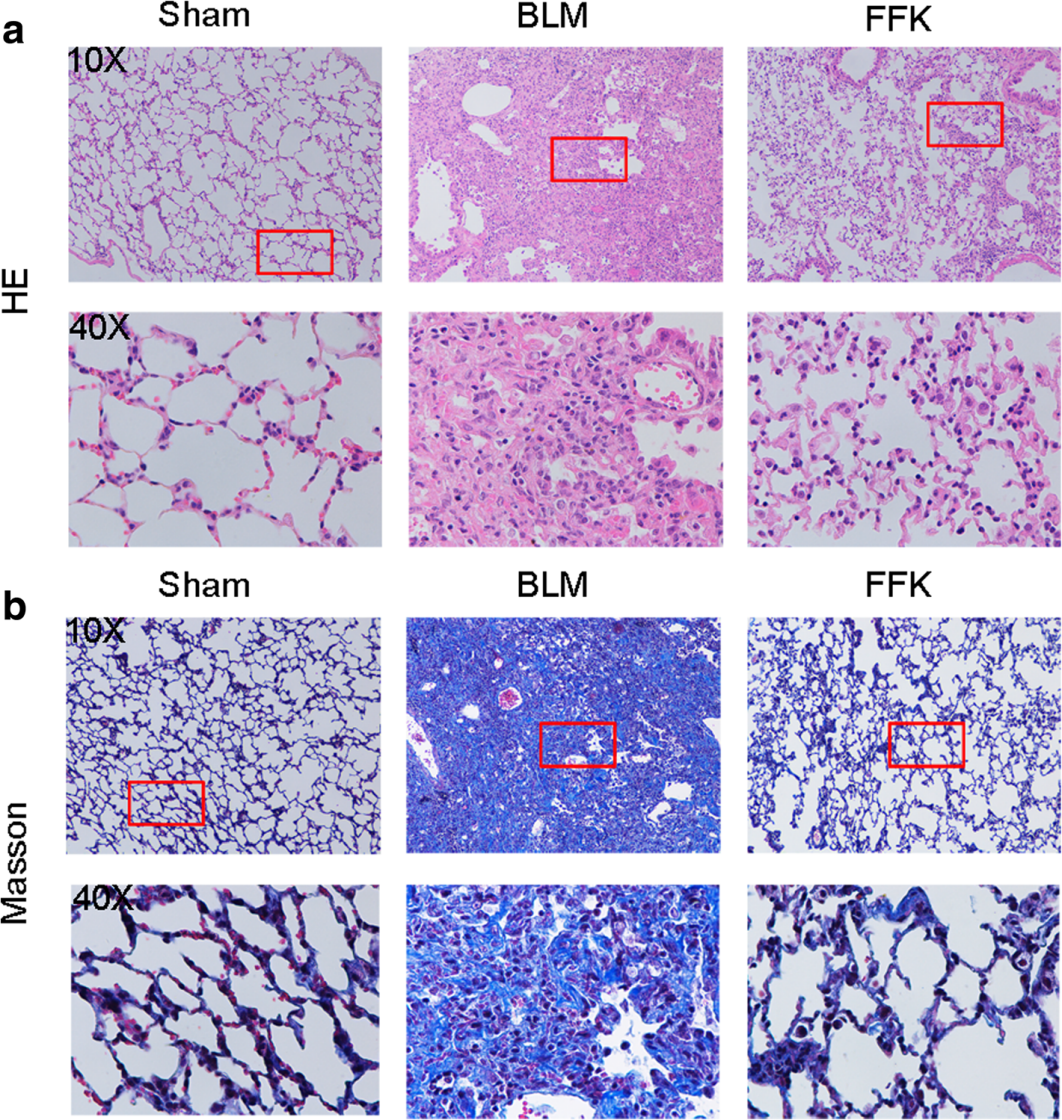
Anti-pulmonary fibrosis of FFK in BLM-treated mice. **a** FFK improved the alveolar structure of mice compared with the BLM group as shown by H&E staining of animal models. **b** FFK inhibited the collagen fibers as shown in Masson’s staining. The color blue represents collagen fibers

**Fig. 2:**
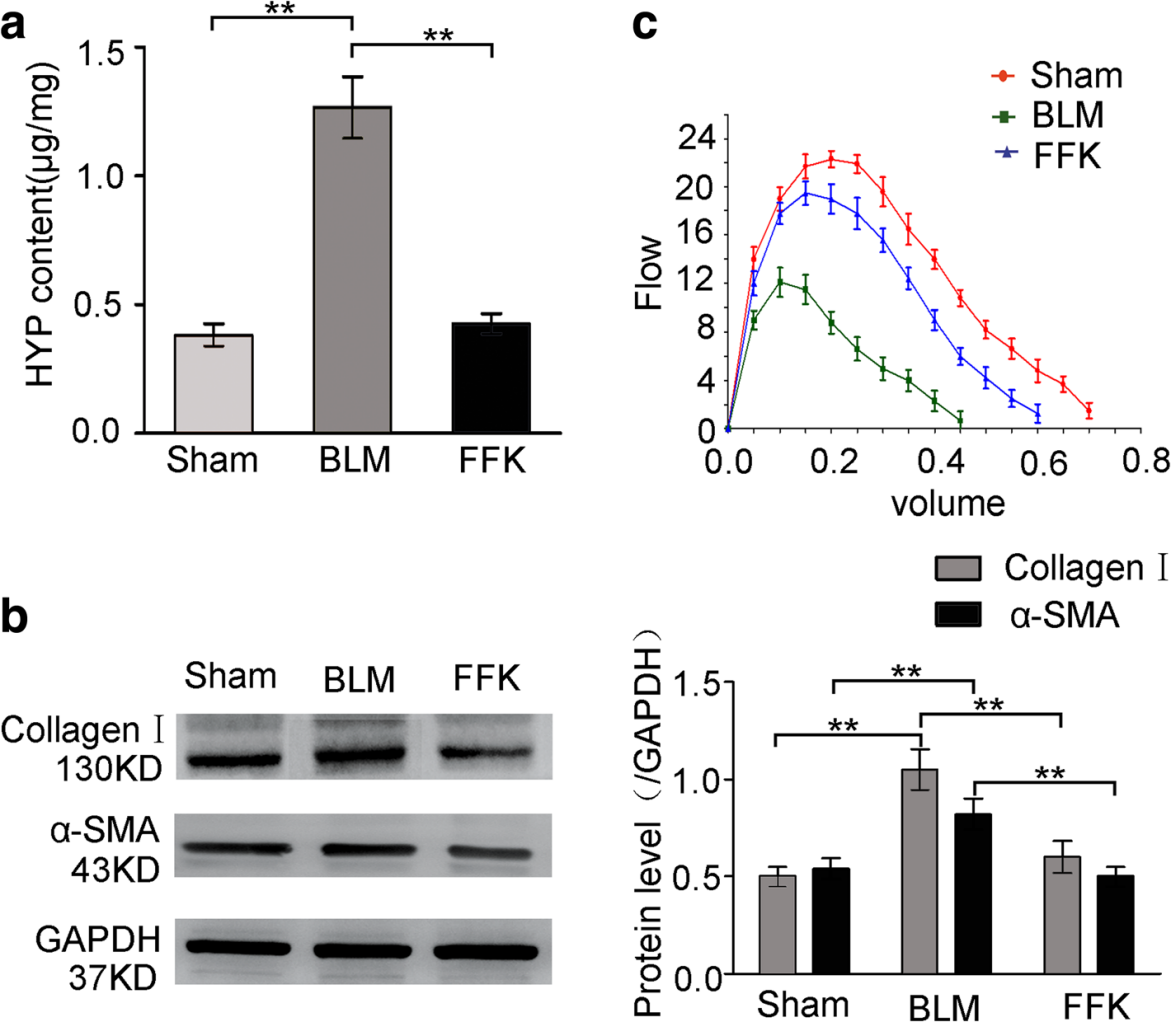
FFK inhibited the indicators of pulmonary fibrosis in BLM-treated mice. **a** FFK remarkably inhibited the hydroxyproline (HYP) level in treated mice compared with that in the BLM-treated mice. **b** FFK significantly inhibited the collagen I and a-SMA expression in the treated mice compared with that in the BLM-treated mice. **c** FFK increased FVC in the treated mice compared with that in the BLM-treated mice. Data are shown as means ± SD, *n* = 6, **P < 0.01

The anti-pulmonary fibrosis effect of FFK was tested in BLM-treated mice. H&E and Masson staining results showed that the BLM group had the thickest alveolar walls among the three groups. Lung mesenchyme in the BLM group showed strong immunohistochemical staining for collagen, thereby indicating the distinctive characteristics of fibroblastic foci. Tissue sections from FFK-treated group showed thinner alveolar walls and lower collagen content than those from the BLM group (Fig. 1). Hydroxyproline, collagen I, and a-SMA, which are key mediators of fibrosis, were also evaluated to confirm the efficacy of FFK. Compared with the sham group, the BLM group showed significantly higher hydroxyproline content, collagen I, and a-SMA expression in lungs. FFK treatment significantly reduced the BLM-induced increase in hydroxyproline content, collagen I, and a-SMA expression (Fig. 2a and b). FFK inhibition on pulmonary fibrosis increased the forced vital capacity (FVC) compared with that of the BLM-treated mice (Fig. 2c).

### Regulation of FFK in pulmonary fibrosis-associated mRNAs

**Fig. 3:**
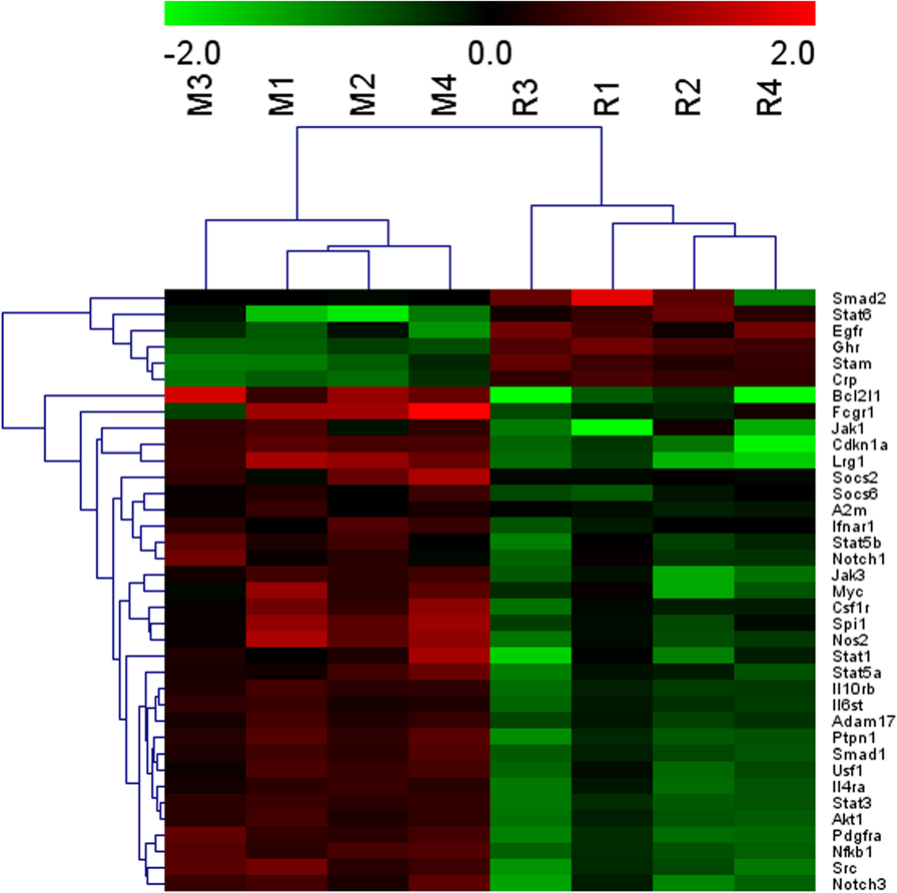
Heatmap of the expression profiles of the differentially expressed mRNAs in mice. Blue to red indicate the low to high expression levels. The column labels at the right represent the differentially expressed mRNAs in the JAK-STAT signaling pathway. M represents the BLM-treated model, and R represents the FFK-treated mice

The differentially expressed mRNAs were evaluated using RNA sequencing to elucidate the anti-pulmonary fibrosis signaling pathway of FFK. The mRNA expression profiles of the FFK-treated and BLM-treated groups were compared according to the RNA sequencing data. Enrichment analysis was conducted to explore the functions of the mRNAs identified in this study. Genes were organized into hierarchical categories to uncover gene regulatory networks based on biological processes, cellular components, and molecular functions. The analysis showed that many dysregulated mRNAs, such as JAK, STAT, a disintegrin metalloproteinase 17 (ADAM17), and Notch, were enriched in the JAK-STAT signaling pathway (Fig. 3).

### Compound–medicine network

Network pharmacology offers a approach of exploring drug targeted genes and identifying potential active ingredients in TCM research. Hence, we confirm the RNA sequencing data by using network pharmacology to analyze the compound–medicine, compound–target, and target–pathway networks of FFK according to the ADME system.

**Fig. 4:**
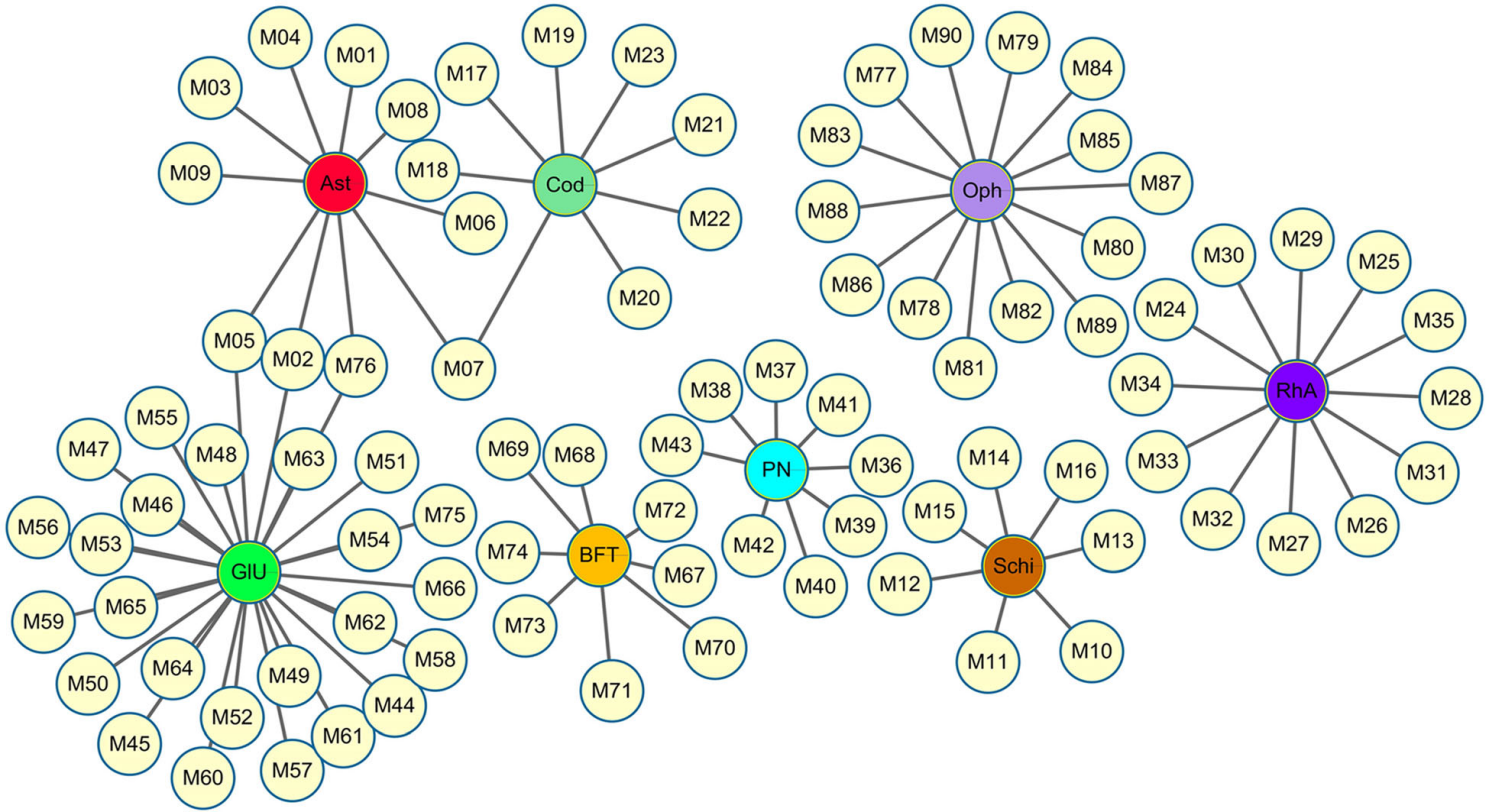
Compound–medicine network. Yellow circular nodes represent the effective chemical compounds from eight herbs in FFK. Rectangle nodes indicate the following herbs: *Astragalus membranaceus* (Ast), *Codonopsis pilosula* (Cod), *Ophiopogon japonicus* (Oph), *Schisandra chinensis* (Schi), *Panax notoginseng* (PN), *Bulbus fritillariae thunbergii* (BFT), *Rhizoma anemarrhenae* (RhA), and *Glycyrrhiza uralensis* (GIU)

First, the bioactive compounds of FFK were assessed in the compound–medicine network. Using the ADME system, 90 bioactive compounds were filtered from eight TCMs in FFK. These components included 10 compounds in *Astragalus*, 8 in *C. pilosula*, 14 in *O. japonicus*, 7 in *S. chinensis*, 8 in *P. notoginseng* (Burk.) F. H. Chen., 8 in *B. fritillariae thunbergii*, 12 in *R. anemarrhenae*, and 27 in *G. uralensis* (Fig. 4).

OB is one of the most important pharmacokinetic parameters among ADME properties and represents the percentage of oral doses that sufficiently produce pharmacological effects. High OB level is often highly considered as therapeutic agents in the development of bioactive molecules. Therefore, evaluation of OB is indispensable in determining pharmacologically active compounds. In this section, OB was applied to prescreen and determine pharmaceutically active compounds in FFK. Table 1 shows 90 chemicals exhibiting high OB. Among these chemicals, four compounds, namely, M05 (formononetin), M07 (nicotinic acid), M66 (calycosin), and M76 (ononin), were duplicated in three herbs. Nicotinic acid was observed in *Astragalus* and *C. pilosula*. Formononetin, calycosin, and ononin were present in *Astragalus* and *G. uralensis*. The four compounds were predicted as the main active compounds of FFK. In our pharmacokinetic assessment of FFK, all the chemical constituents of FFK were determined using high-performance liquid chromatography. Six compounds, including formononetin, calycosin, ononin, mangiferin, calycosin-7-O-glucoside, and liquiritin, were separated from FFK. Among these compounds, formononetin, calycosin, and ononin were predicted from the compound–medicine network [22].

**Table 1:** Ninety bioactive compounds filtered through the ADME system in PRM

ID	Compound	OB
M01	Vanillic acid	35.4723531931
M02	Jaranol	50.8288167701
M03	Isoferulic acid	50.8264760705
M04	Bifendate	31.0978239059
M05	Formononetin	69.6738806088
M06	Caffeate	54.9705395881
M07	Nicotinic acid	47.6452927768
M08	1,7-dihydroxy-3,9-dimethoxy pterocarpene	39.0454111203
M09	Prolinum	77.5746812914
M10	Longikaurin A	47.7221498383
M11	Deoxyharringtonine	39.2744398817
M12	Arnebin 7	73.8482146
M13	Schizandrer B	30.70577053
M14	Gomisin-A	30.6937534295
M15	Gomisin R	34.84255546
M16	Wyerone	79.2373604652
M17	6-methylolpyridin-3-ol	47.5280504103
M18	Perlolyrine	65.94775259
M19	Daturilin	50.3651347237
M20	Glycitein	50.4789136567
M21	Tangshenoside II_qt	51.7225585616
M22	11-hydroxyrankinidine	40.002764
M23	Codopiloic acid	57.4989613831
M24	Asperglaucide	58.0162962407
M25	Tingenone	12.0351082251
M26	Sarsapogenine	17.4080449729
M27	Smilagenin	14.1527227832
M28	Anhydroicaritin	45.4119342096
M29	Gitogenin	16.1298377929
M30	Timosaponin A III_qt	14.6470444657
M31	Timosaponin A-1_qt	13.2763899535
M32	Anemarsaponin B_qt	9.79986290093
M33	(Z)-3-(4-hydroxy-3-methoxy-phenyl)-n-[2-(4-hydroxyphenyl)ethyl]acrylamide	118.347748464
M34	Diosgenin	80.8779249091
M35	Coumaroyltyramine	112.901574879
M36	(+)-ledol	16.9554765285
M37	(−)-alpha-cedrene	55.56099313
M38	Alloaromadedrene	53.4613596861
M39	Panaxydol	61.6665994071
M40	Panaxytriol	33.75825582
M41	α-cyperene	51.1046007793
M42	Hepanal	53.8331756683
M43	Quercetin	46.4333481195
M44	Lupiwighteone	51.6356918063
M45	Glyasperin F	75.8368001254
M46	Isotrifoliol	31.9447872421
M47	(E)-1-(2,4-dihydroxyphenyl)-3-(2,2-dimethylchromen-6-yl)prop-2-en-1-one	39.6168553714
M48	Kanzonols W	50.48007599
M49	Glepidotin A	44.7218746499
M50	Glepidotin B	64.462923857
M51	Gancaonin B	48.7944020143
M52	3-(3,4-dihydroxyphenyl)-5,7-dihydroxy-8-(3-methylbut-2-enyl)chromone	66.37125046
M53	2-(3,4-dihydroxyphenyl)-5,7-dihydroxy-6-(3-methylbut-2-enyl)chromone	44.15196126
M54	Licoisoflavone	41.6102188517
M55	Licoisoflavone B	38.9287088773
M56	Licoisoflavanone	52.4662470622
M57	Glyzaglabrin	61.0688863093
M58	Glabrene	46.266857212
M59	1,3-dihydroxy-9-methoxy-6-benzofurano[3,2-c]chromenone	48.1415423489
M60	Eurycarpin A	43.2772842533
M61	6-prenylated eriodictyol	39.2238301837
M62	7-acetoxy-2-methylisoflavone	38.9233310489
M63	Vestitol	74.65518912
M64	Glyasperins M	72.6708098439
M65	Licoagroisoflavone	57.2822409825
M66	Calycosin	47.7518278266
M67	Pelargonidin	37.9883123298
M68	Peimisine	57.4023933024
M69	Zhebeiresinol	58.7205344913
M70	Ziebeimine	64.2465779173
M71	Verticine	17.4196730712
M72	6-methoxyl-2-acetyl-3-methyl-1,4-naphthoquinone-8-o-beta-d-glucopyranoside_qt	19.8705551162
M73	OSI-2040	14.6514295106
M74	Peiminoside_qt	11.7527635822
M75	Odoratin	7.82764281419
M76	Ononin	11.5220564862
M77	6-aldehydo-isoophipogonone A	
M78	6-aldehydo-isoophipogonone B	
M79	n-trans-feruloyltyramine	
M80	Ophiopogonanone A	
M81	Ophiopogon B	
M82	Ophiopogonin A	
M83	Ophiopogonin B	
M84	Ophiopogonone A	
M85	Ophiopogonone B	
M86	Orchinol	
M87	Guanosine	
M88	Stigmasterol	
M89	Diosgenin	
M90	Uridine	

Calycosin affects proliferation, metastatic recurrence, and metastasis of A549 cells by regulating the protein expression levels of matrix metalloproteinases (MMPs) through the inhibition of the protein kinase C alpha/extracellular signal-regulated kinase 1/2 pathway [23]. Calycosin-7-O-β-D-glucoside can promote oxidative stress-induced cytoskeleton reorganization through the integrin-linked kinase signaling pathway in vascular endothelial cells [24]. Formononetin, specifically 7-hydroxy-3-(4-methoxyphenyl)-4 h-chromen-4-one, is the aglycone that was hydrolyzed in vivo from ononin. This aglycone can inhibit the inflammation of lipopolysaccharide-induced acute lung injury in mice; this type of injury is associated with the induced expression of peroxisome proliferator-activated receptor-γ and the suppressed proliferation of human non-small cell lung cancer through cell cycle arrest and apoptosis [25, 26]. Formononetin can also inhibit the migration and invasion of breast cancer cells by suppressing MMP2 and MMP9 through phosphoinositide 3-kinase/protein kinase B (PI3K–Akt) signaling pathways [27].

### Compound–target network

**Fig. 5:**
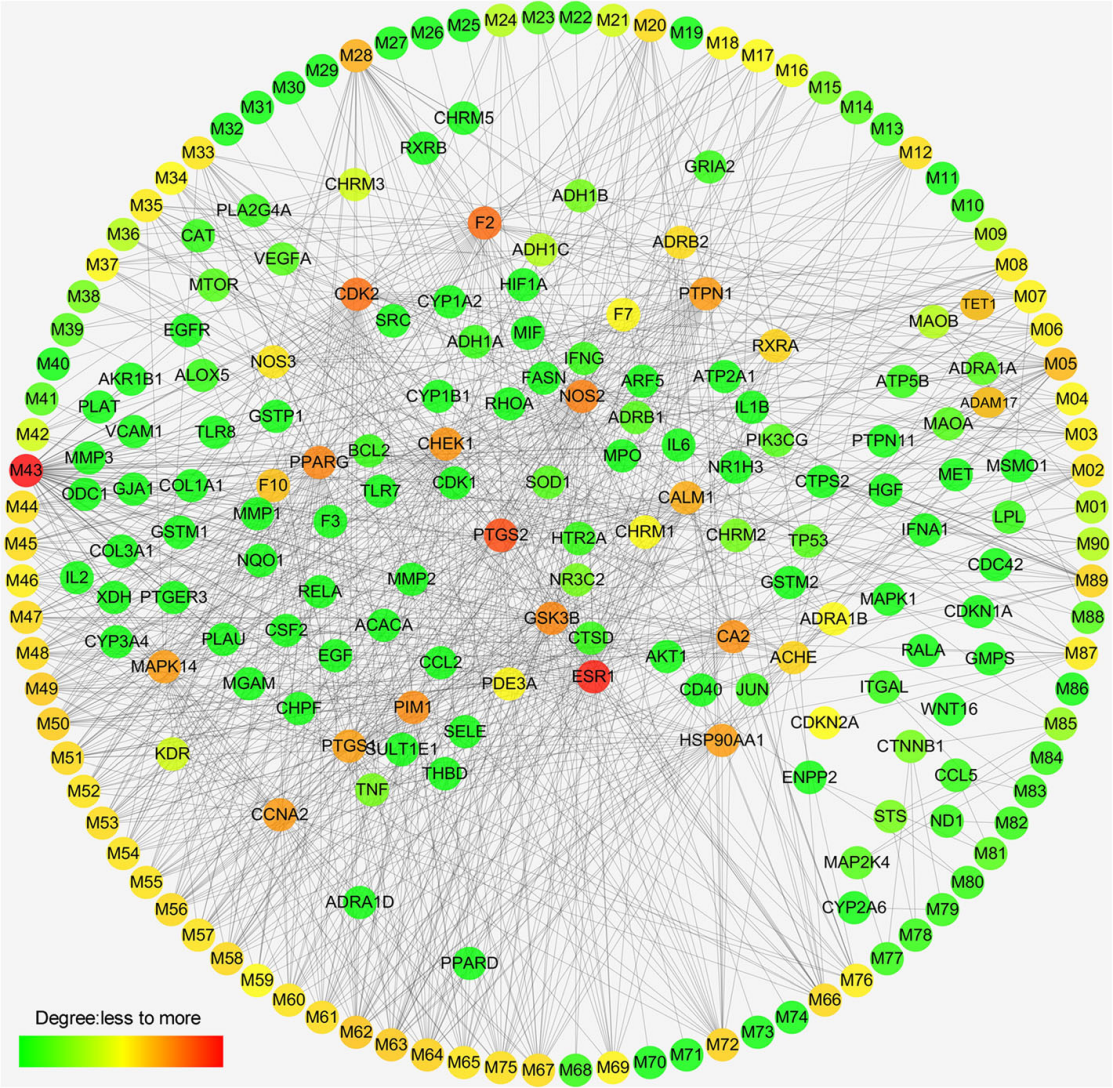
Compound–target network. Circular nodes represent the effective chemical compounds, and rectangle nodes represent the therapeutic target genes

After ADME screening, a bipartite graph for compound–target network (Fig. 5) was constructed for the 90 compounds by connecting them to 129 potential targets through 914 interactions. Compound–target network analysis was performed by evaluating the degrees of nodes, resulting in average degrees of 10.16 and 7.20 per compound and target, respectively. A high number (>97%) of representative active compounds exhibited degrees higher than the average values. These compounds were considered clinically valid. Among these active compounds, M43 (quercetin) in *P. notoginseng* exhibited the highest number of interactions with various targets; such interactions are associated with multiple pathways, which are involved in inflammation and oxidative stress and prevent pulmonary fibrosis and lung injury [28, 29].

Among the 129 targets, 83 were associated with pathways related to progression of pulmonary fibrosis, and the remaining 46 were associated with the pathophysiology of the disease. For example, monoamine oxidase (MAO) B and MAO A are associated with histidine metabolism. Prostaglandin G/H synthase 1 (PTGS1), arachidonate 5-lipoxygenase, and several other targets are associated with arachidonic acid metabolism, which is probably associated with progression of inflammation related to pulmonary fibrosis. Among these targets, estrogen receptor possesses the highest number of connected ingredients, which are associated with endocrine and other factor-regulated calcium reabsorption processes. PTGS2 and thrombin play pivotal roles in inflammatory and tissue repair responses through fibrin generation and activation by coagulation pathways in acute and fibrotic lung injury [30]. Several other targets are associated with other diseases. For example, retinoid X receptor beta and tumor protein *p53* RELA are associated with tuberculosis and small-cell lung cancer.

### Target–pathway network

**Fig. 6:**
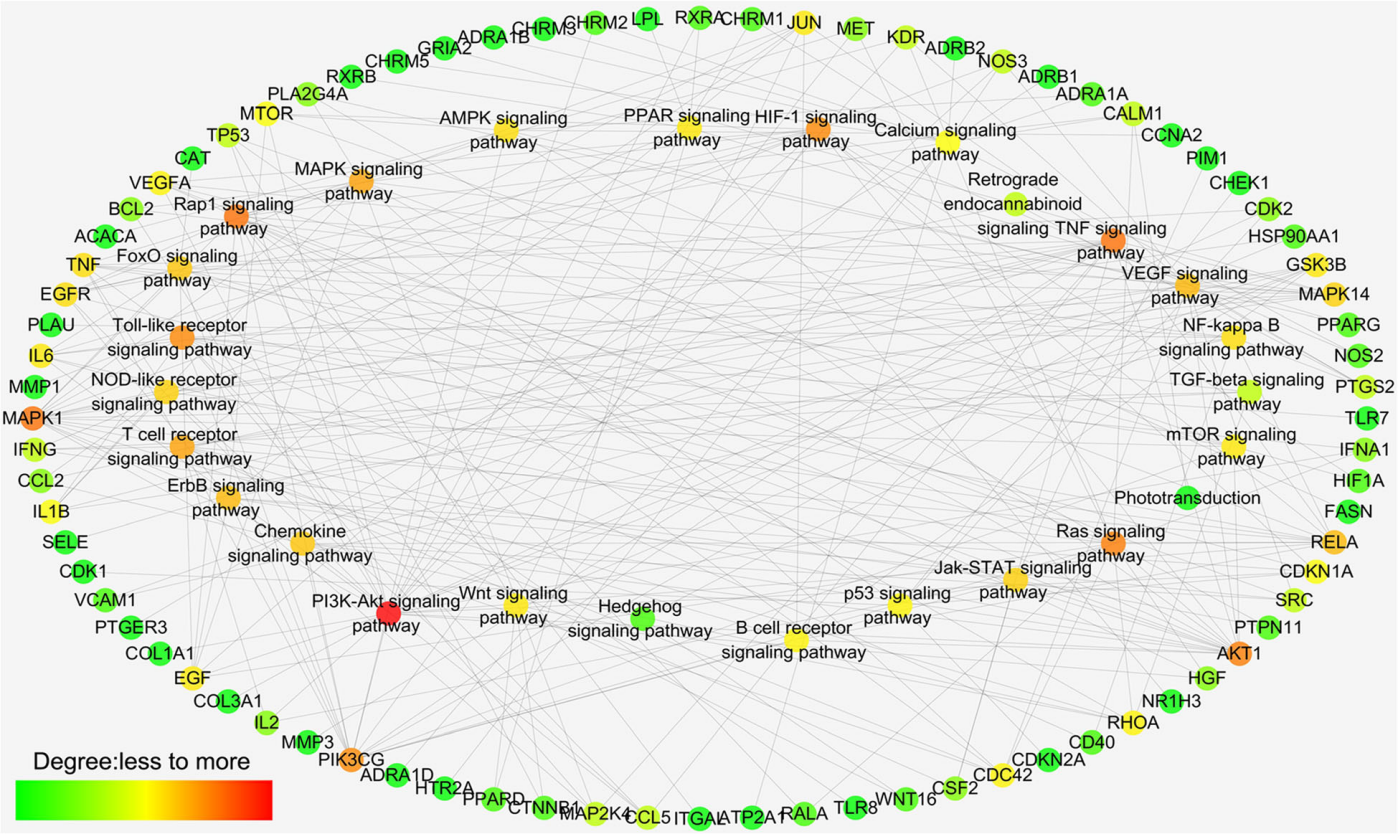
Target–pathway network. Network of 26 signaling pathways (rectangle nodes) connected to 80 target genes (circular nodes)

We applied a target-based approach to probe pathways that are possibly involved in therapeutic actions, build the target–pathway network, and indicate interactions between targets and pulmonary fibrosis therapy-associated pathways (Fig. 6). Eighty targets obtained were further mapped onto 26 pathways, showing average degrees of 3.35 and 10.30 per target and pathway, respectively. Pathways such as PI3K–Akt, calcium, nucleotide oligomerization domain (NOD)-like receptor, and mechanistic target of rapamycin (mTOR) signaling pathways, were intensively connected to the targets. Drugs may induce their antifibrotic effects through these pathways, which were already verified and widely used for pulmonary fibrosis therapies [31]. For instance, the NOD-like receptor-family protein 3-inflammasome pathway releases proinflammatory cytokines and interleukin-1β in the lungs. This receptor is also involved in experimental collagen deposition and development of pulmonary fibrosis [32]. mTOR overactivation in alveolar epithelial cells and compromised autophagy in lungs contribute to the pathogenesis of pulmonary fibrosis [33]. BLM promotes the development of inflammation, which results in severe pulmonary fibrosis and increased TGF-β1, Smad3, and signal transducer and activator of transcription (STAT). A possible pathway exists for mouse pulmonary fibrosis model through the JAK-STAT pathway [34].

### Involvement of JAK-STAT in the antifibrotic pathway of FFK

**Fig. 7:**
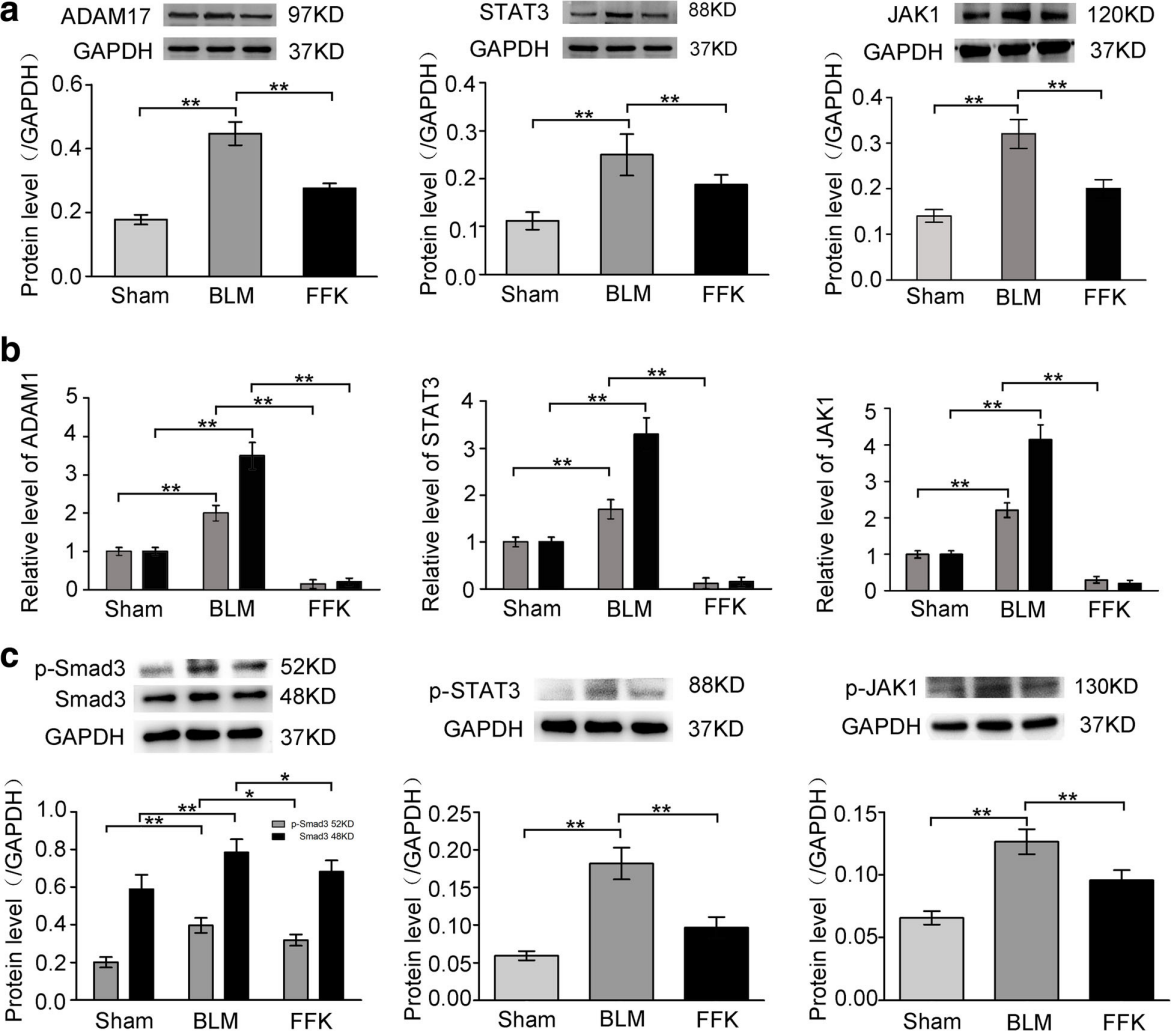
Expression of JAK1, STAT3 and ADAM17 were tested in mice. **a** Protein expression level of JAK1, STAT3 and ADAM17 by using Western blot. FFK remarkably decreased JAK1, STAT3 and ADAM17 protein expressions compared with BLM. **b** mRNA expression level of JAK1, STAT3 and ADAM17 by using qRT-PCR and RNA-sequencing. FFK remarkably decreased JAK1, STAT3 and ADAM17 mRNA expressions compared with BLM. **c** Western blot showed that FFK reduced p-SMAD3, p-STAT3 and p-JAK1. Data are shown as means ± SD, *n* = 6, **P < 0.01

To confirm the JAK-STAT signaling pathway involvement in the antifibrotic pathway of FFK, JAK1, STAT3 and ADAM17 were selected as representative genes by combining the data of RNA sequencing and network pharmacology. qRT-PCR and Western blot analysis showed that JAK1, STAT3 and ADAM17 levels increased in the pulmonary fibrosis model in vivo, whereas FFK remarkably decreased JAK1, STAT3 and ADAM17 expression (Fig. 7a and b). Protein phosphorylation plays important roles in cell signal transduction, so p-SMAD3, p-STAT3 and p-JAK1 were further detected by using Western blot method. The results demonstrated that FFK also reduced these phosphorylated proteins expression (Fig. 7c).

### Analysis of co-expressed long noncoding RNA (lncRNA) with JAK-STAT pathway

**Fig. 8:**
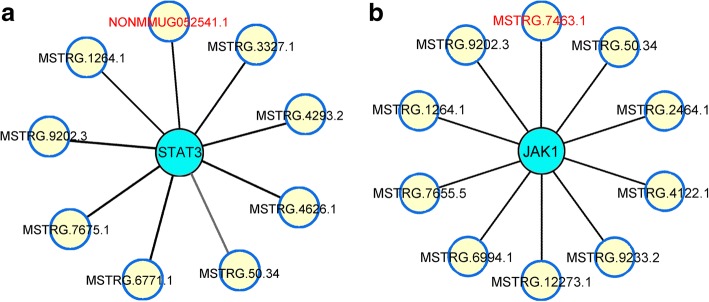
Co-expressed lncRNAs with JAK1 and STAT3. **a** Nine lncRNAs, NONMMUG052541.1, MSTRG.1264.1, MSTRG.3327.1 MSTRG.4293.2, MSTRG.4626.1, MSTRG.50.34, MSTRG.6771.1, MSTRG.7675.1, MSTRG.9202.3, co-expressed with STAT3. Among these lncRNAs, NONMMUG052541.1 had the highest co-expressed degrees with STAT3. **b** Ten lncRNAs, MSTRG.7463.1, MSTRG.50.34, MSTRG.2464.1, MSTRG.4122.1, MSTRG.9233.2, MSTRG.12273.1, MSTRG.6994.1, MSTRG.7655.5, MSTRG.1264.1, MSTRG.9202.3, co-expressed with JAK1. Among these lncRNAs, MSTRG.7463.1 had the highest co-expressed degrees with JAK1

lncRNA is the upstream factor of mRNA that regulates its function. Based on the RNA sequencing data, JAK1, STAT3, ADAM17, and their co-expressed lncRNAs in the FFK-treated group were analyzed and compared with those in the BLM-treated group. The data showed that no lncRNA could co-express with ADAM17. However, nine lncRNAs could co-express with STAT3, ten with JAK1. Among these lncRNAs, lncRNA-MSTRG7464.1 and lncRNA-NONMMUG052541.1 have the highest co-expressed degrees with JAK1 and STAT3. The value of co-expressed degrees are 2.13 and 1.97, respectively (Fig. 8).

## Discussion

In this study, the effect of FFK on BLM-induced lung fibrogenesis and its anti-fibrotic signaling pathway were investigated in mice. Our results showed that FFK significantly decreased the alveolar wall thickness and collagen fiber formation. These anti-fibrotic effects of FFK may be mediated by blocking the JAK-STAT signaling pathway.

Pulmonary fibrosis is an interstitial lung disease associated with aging and characterized by histopathological patterns of common interstitial pneumonia [35]. Epithelial cells, resident fibroblasts, and immune cells communicate with one another in a complex mechanism, which evolves to initiate and promote fibrosis [36]. However, no current therapeutic approach can treat the disease. Alternative herbal medicines, which are characterized as multiple compounds and targets, exhibit advantages in the treatment of pulmonary fibrosis. Unfortunately, the detailed molecular signaling pathway remains intractable because the potential targets and active substances of herbs are difficult to identify and analyze [37, 38]. High-throughput technologies, such as RNA sequencing and network pharmacology, offer approaches for exploring drug targets and identifying potential active ingredients in TCM [39]. In this study, the targeted genes and signaling pathways in FFK treatment were analyzed by using RNA sequencing and network pharmacology. The RNA sequencing findings showed that FFK can ameliorate pulmonary fibrosis through multi-genes and multi-pathways. Particular genes, including JAK-STAT family members (Jak, Stat), JAK-STAT signaling regulators (Socs, Bcl2l1), and STAT-interacting transcription factors and regulators (Smad, Nfkb1), are some of the most notable components of the JAK-STAT signaling pathway.

An integrated systematic network pharmacological method was further utilized to explore the complex therapeutic mechanism of FFK and confirm the RNA sequencing data. The results showed that FFK played an important role in oxidative phosphorylation, apoptosis, inflammation, and regulation of autophagy, cell adhesion molecules, and extracellular matrix–receptor interaction, which are regulated by JAK-STAT, PI3K–Akt, TGF-β, and other signaling pathways. As a TCM with combined anti-inflammatory, anti-oxidant, and anti-fibrotic effects, FFK exhibits therapeutic potential for pulmonary fibrosis.

The influence of FFK on JAK-STAT signaling pathway was further examined based on these results. ADAM17 is a molecular switch that controls immune responses, tissue regeneration, and cancer development [40]. This gene is upregulated in tumor cells almost ubiquitously [41] and is rarely reported in fibrotic diseases. Our previous study showed that ADAM17 is a target gene of miR-708-3p, which can induce aberrant fibrosis via STAT3-dependence on ADAM17 signaling pathways [42]. In the current study, the expression level of JAK1, STAT3 and ADAM17 decreased in FFK treatment group compared to BLM treatment group, thus indicating the possible pathways implicated in lung fibrosis as targets of therapeutic attempts. What is the rationale behind FFK mediated JAK-STAT signaling pathway? Considering the mechanism diversity and complexity for drug action, protein phosphorylation was chose to demonstrate how FFK regulated JAK-STAT signaling pathway. Protein phosphorylation, as an extremely important protein posttranslational modification, participates in almost all life activities and plays important roles in cell signal transduction. Our result showed that FFK decreased the levels of p-SMAD3, p-JAK1, p-STAT3. We inferred that FFK blocked JAK-STAT pathway through regulating the relevant protein phosphorylation. Certainly, cellular transmembrane signal transduction is in a complicated way, experiments will be designed to determine the FFK regulation on JAK-STAT pathway for future research.

High-throughput technologies revealed that only 2% of the transcribed genome codes are attributed to proteins. With the wide-scale adoption of high-throughput sequencing techniques, these noncoding RNAs were described as a novel drug targets or biomarkers of various diseases. These RNAs also represent a potential research hotspot in disease treatment. Among the various types of noncoding RNAs, lncRNA has attracted increasing attention [43]. Here, we revealed that the lncRNAs target JAK-STAT signaling pathway regulate the anti-pulmonary fibrosis mechanism of FFK. Certainly, further experiments should be designed to determine the relationship between FFK and lncRNAs-mediated pulmonary fibrosis for future research.

## Conclusion

This work studied the anti-pulmonary fibrosis and signaling pathways of FFK. Results can remarkably explain that FFK showed efficacy as pulmonary fibrosis treatment through multi-genes and multi-pathways. The targeted genes in JAK-STAT signaling pathway are some of the most notable components of these multi-genes and multi-pathways. We hope provide the theoretical and experimental basis for the clinical application of FFK for lung fibrosis treatment. We also hope to provide a new idea for the study of TCM.
